# Environmental factors influencing tick densities over seven years in a French suburban forest

**DOI:** 10.1186/s13071-016-1591-5

**Published:** 2016-05-27

**Authors:** Richard E. L. Paul, Martine Cote, Evelyne Le Naour, Sarah I. Bonnet

**Affiliations:** Department of Genome and Genetics, Institut Pasteur, Unité de Génétique Fonctionnelle des Maladies Infectieuses, 28 rue du docteur Roux, 75724 Paris, France; Centre National de la Recherche Scientifique, URA3012, 28 rue du docteur Roux, 75724 Paris, France; UMR BIPAR INRA-ANSES-ENVA, 14 rue Pierre et Marie Curie, 94701 Maisons-Alfort cedex, France

**Keywords:** Climate impact, *Ixodes ricinus*, Longitudinal study, Peri-urban forest, Ticks, Tick-borne pathogens, Tick density

## Abstract

**Background:**

Worldwide changes in socio-economic and environmental factors and the global climate are recognised causes of variation in tick distribution and density. Thus it is of great importance that new studies address the changing risk of infection for exposed populations. In Europe, *Ixodes ricinus* ticks are the most common vectors of several pathogens impacting veterinary and public health that have colonised suburban habitats.

**Methods:**

This study aimed to evaluate longitudinal *I. ricinus* questing densities and infection rates over 7 years in a French suburban forested area with high human population density. Ticks were collected in spring yearly between 2008 and 2014 and, out of a total of 8594 collected *I. ricinus*, a representative subset of adult females (*n* = 259) were individually examined for the presence of several pathogens via PCR.

**Results:**

Nymph densities peaked in 2009–2011, and then declined in 2012–2014. Changes in monthly temperature only had a modest impact on this variation. In contrast, analysis revealed a complex intra-annual relationship between mean nymph density and both concurrent and lagged mean monthly temperatures. The following pathogens were detected in the studied area: *Anaplasma phagocytophilum*, *Rickettsia helvetica*, *Babesia venatorum* and *B. divergens*, *Francisella tularensis*, *Borrelia miyamotoi*, *B. afzelii/valaisiana*, *B. garinii/lusitaniae* and *Bartonella* spp.

**Conclusion:**

Our findings reinforce the conclusion that ticks are important vectors of pathogenic microorganisms in suburban forests and suggest that despite complex intra-annual relationships between tick densities and temperature, there is no evidence for a climate-associated increase in infection risk over the 7-year period. Rather, tick densities are likely to be strongly influenced by population density fluctuations in vertebrate host species and wildlife management. Further detailed studies on the impact of climate change on tick population densities are required.

**Electronic supplementary material:**

The online version of this article (doi:10.1186/s13071-016-1591-5) contains supplementary material, which is available to authorized users.

## Background

Ticks are ranked second after mosquitoes as the most frequent vectors of pathogens causing human disease, and are the most important vectors of pathogens affecting both domestic and wild animals worldwide [1]. The geographical distribution of several tick species is believed to have expanded due to both the intensification of human and animal activity, and socio-economic and environmental changes. Furthermore, ticks can easily spread and colonise novel regions via host movements, either during domestic animal transport or via bird migration, which is perfectly illustrated by the recent *Rhipicephalus microplus* colonisation of East-African territories [2]. In addition, recent transcriptomic studies of *Ixodes ricinus* ticks from eastern France using next-generation sequencing techniques, have led to the identification of unexpected bacteria and parasites, some of them pathogenic for humans or animals [3, 4]. Thus the emergence or re-emergence of tick-borne diseases (TBD) is a growing concern [1, 5], and TBD incidence is on the rise in several European countries [6], highlighting the need for increased surveillance of tick populations and any pathogens they may transmit.

In Europe, *I. ricinus* is the most abundant and widespread tick species and the vector of several TBDs of medical and veterinary importance. These include Lyme disease, tick-borne encephalitis and rickettsiosis in humans, and babesiosis and anaplasmosis in livestock [7]. Indeed, *I. ricinus* feed on a remarkably large variety of vertebrate hosts and frequently bite humans [8]. Consequently, this obligate hematophagous acarine transmits an extensive variety of pathogenic organisms that can cause mild to severe illness and occasional death in humans, domesticated animals and wildlife [8]. The primary habitat of *I. ricinus* is scrubland and deciduous or mixed forests, which host a high abundance of small, medium and large wild vertebrate hosts. However, the distribution of *I. ricinus* has significantly expanded over recent decades due to several factors, among which, changes in land cover and use, and forestry and wildlife management, are thought to be the most significant [9]. Suburban locations proximate to natural woodlands have expanded the natural habitat suitable for *I. ricinus*, and concomitantly, the prevalence of human infections due to tick-borne pathogens has increased at both urban and suburban sites in many European countries [8]. These observations are also due in part to increased sensitivity of the diagnostic methods, as well as the more frequent contact between ticks, wildlife and humans, favoured by the rise in outdoor recreational activities [8].

Despite concerns relating to increased numbers of ticks and prevalence of tick-borne pathogens, relatively few studies have evaluated longitudinal *I. ricinus* densities [10–15], or whether there are concomitant changes in pathogen prevalence. Both abiotic (temperature, rainfall and relative humidity) and biotic (animal abundance and herbaceous strata) influence tick densities and activity locally [15–17]. Many studies have demonstrated the importance of both current and lagged monthly temperatures on questing tick densities [10, 13 15, 17]. Tick activity is considerably influenced by temperature, with clear temperature-driven seasonal activity in temperate regions. The herbaceous strata determine fine scale relative humidity and, because ticks are highly sensitive to desiccation, the herbaceous strata will thus also delimit questing behavior. Although these factors impact upon tick questing activity at the very local scale, use of remote sensing technology and larger scale measures of climate factors potentially enable generation of spatio-temporal risk maps for tick exposure at a scale more adapted to public health use [18]. Benefiting from a 7-year longitudinal study, we examine the relationship between variation in climate factors and *I. ricinus* questing densities and assess any associated inter-annual changes of infection risk through human and animal pathogen detection.

## Methods

### Study area and tick collection

**Fig. 1:**
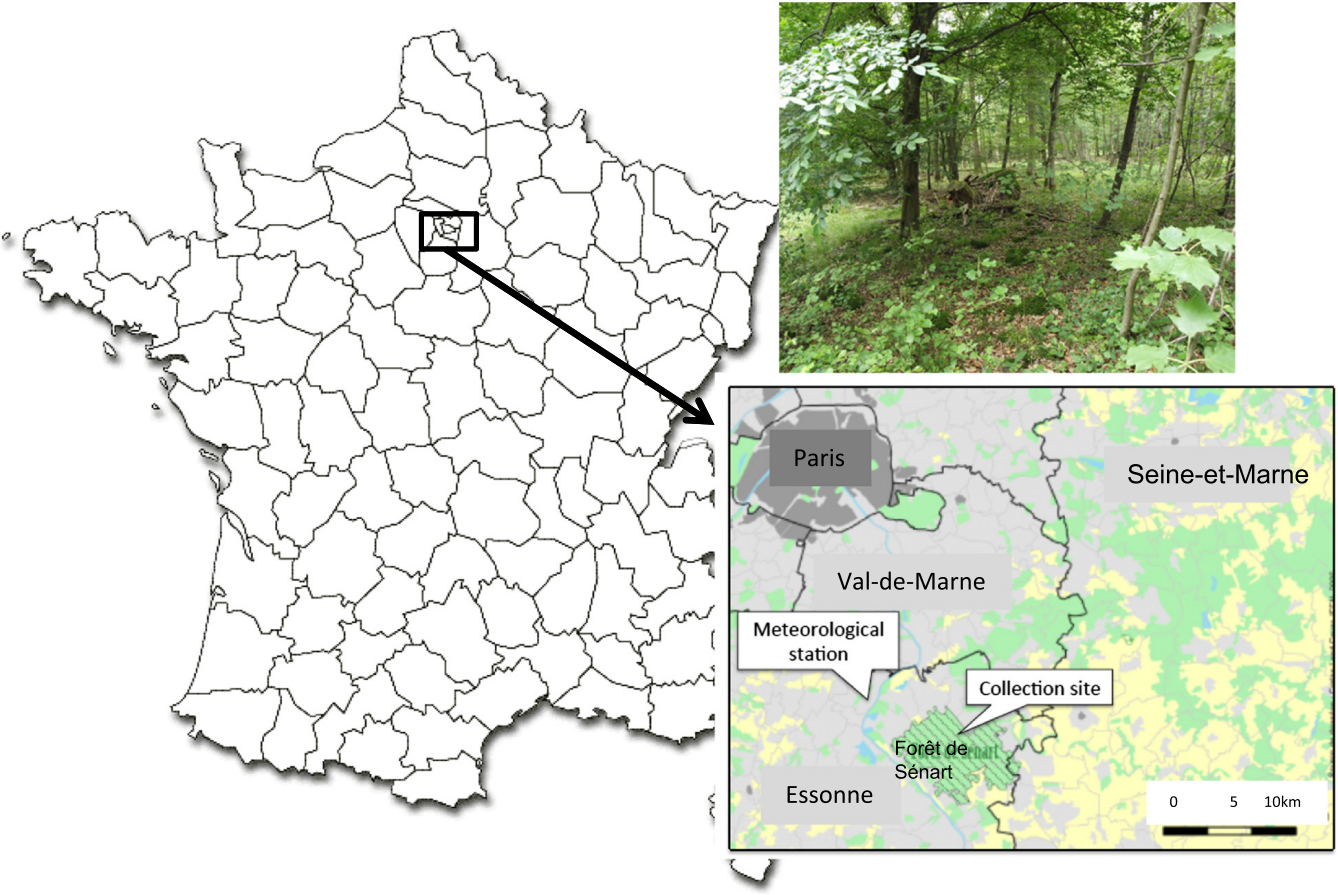
Map of France showing the location and habitat of the monitoring area. Inserts show the location of the tick collection site and the meteorological station, and representative habitat of the collection site in the Senart forest

Questing ticks (nymphs and adults) were collected by flagging vegetation within a 100 × 200 m plot of land located in the north of the Sénart Forest (48°40′N, 2°29′E), and representative of the forest regarding the fauna, flora and human recreational activities (Fig. 1). The Sénart forest is one of the oldest and largest (30 km^2^) forests within the Ile-de-France region (France), which is one of the most populated metropolitan areas in Europe (974 inhabitants per km^2^). This forest is located in the southern Parisian metropolitan belt, and is surrounded by urban zones (Fig. 1). It is predominantly deciduous and hosts abundant numbers of large mammals (wild boar, roe deer), small rodents and birds, creating an ideal environment for ticks. Because of its location and the wide variety of available recreational activities, the forest is visited by over three million people annually [19].

Ticks were collected in spring (from March to June) yearly from 2008 to 2014. Vegetation type and canopy cover within the studied area remained constant throughout the study. Flagging was carried out by drawing a 1 m^2^ cotton cloth over the vegetation, and was performed between one to four times each month (Table 1) [20]. Ticks were collected from 10:00 to 17:00 CEST on days without rain. For every collection event, the temperature, the time and duration of collection (total of 79 h with a mean of 11.3 h per spring), the number of collectors (from 4 to 7 people at each collection time point, mean 4.3), and the number and species of collected ticks were recorded. All specimens, returned alive to the laboratory, were identified to the species level using taxonomic keys [21], and categorised by sex and life stage. Tick densities were estimated as ticks per collector per hour. Throughout the entire collection period from 2008 to 2014, meteorological statistics (monthly minimum, mean and maximum temperature, the number of frost days/month (minimum temperature below 0 °C), the number of ice days/month (maximum temp below 0 °C), and monthly precipitation data, were obtained from the Orly station (48°44′N, 2°24′E), situated at approximately 10 km from the study area (Fig. 1), via the *Météo-France* website (http://www.meteo60.fr/previsions-meteo-france-orly.html.

**Table 1:** *Ixodes ricinus* collections in Sénart Forest (France) summarised by month from spring 2008 to spring 2014, and the corresponding meteorological data

Year	Month	Number of collections	Ppn (mm)	Tmean (°C)	Number of nymphs	Number of adult females	Number of adult males	Total number of ticks	Tick number/collector/hour
2008	May	3	51.2	16.8	263	65	88	416	24
2008	Jun	1	19	17.9	115	38	49	202	10
2009	Apr	4	44.4	12.7	1669	249	270	2188	50.25
2009	Jun	2	54.8	17.3	137	31	34	202	9.75
2010	May	1	37.4	13.1	425	116	98	639	26.25
2010	Jun	1	81.6	18.5	433	78	66	577	15.75
2011	Mar	1	13.4	9.2	153	33	22	208	6
2011	Apr	1	5.8	14.3	410	17	28	455	17.5
2011	May	2	4.2	16.2	302	71	81	454	13.5
2012	Mar	2	16.6	10.3	158	28	23	209	15
2012	Apr	1	65.6	9.5	66	38	31	135	6
2012	May	1	54.6	15.4	85	24	31	140	8
2012	Jun	2	41.4	16.9	195	121	159	475	22
2013	Apr	2	26.8	10.2	99	40	23	162	15
2013	May	1	77.6	12.1	100	11	9	120	12.5
2013	Jun	3	97.2	17	910	61	59	1,030	37.25
2014	Mar	2	9.4	9.3	290	15	8	313	21.5
2014	Apr	1	48.6	12.4	93	14	22	129	7
2014	May	1	88.8	14	66	20	18	104	5.25
2014	Jun	2	63.6	17.8	325	63	48	436	15.5

*Abbreviations*: *Ppn* precipitation (mm); *Tmean* mean temperature recorded on site

In 2008, individual questing female ticks were screened for pathogens [22], whereas from 2009 to 2014, pathogen detection was performed using representative samples consisting of post-laying carcasses of female ticks that had completed feeding on a laboratory rabbit, as previously described [23], in order to include the non-infected progenies in our tick colony. Lack of financial and human resources restricted unfortunately our capacity to detect pathogens in nymphal ticks, and to evaluate prevalence for this tick life stage.

### DNA extraction and PCR amplification

Just after collection or laying, ticks were individually crushed in a bead beater (mixer mill MM301, Qiagen, Hilden, Germany) as previously described [24]. DNA was extracted using the Nucleospin Tissue kit according to the manufacturer’s instructions (Macherey-Nagel, Duren, Germany) and eluted in a final volume of 50 μl. DNA extracts were then stored at -20 °C until used. Successful DNA extraction was confirmed in all samples by polymerase chain reaction (PCR) amplification of the *16S rRNA* mitochondrial gene using tick-specific primers TQ16S + 1 F (5′-CTG CTC AAT GAT TTT TTA AAT TGC TGT GG-3′) and TQ16S-2R (5′-ACG CTG TTA TCC CTA GAG-3′), as described previously [25].

The presence of *Borrelia burgdorferi* (*s.l*.), *Borrelia miyamotoi*, *Anaplasma* spp., *Candidatus* Midichloria mitochondrii, SFG *Rickettsia* spp., *Babesia* spp., *Francisella tularensis* and *Bartonella* spp. DNA in total tick DNA extracts was analysed via specific PCRs for each microorganism, as described previously [22]. All PCRs were individually performed in a MyCycler thermocycler (Bio-Rad, Strasbourg, France). Each reaction was carried out in 25 μl volume containing 0.5 μmol/μl of each primer, 2.5 mmol/l of each dNTP, 2.5 μl 10× PCR buffer, 1 U Taq DNA polymerase (Takara Biomedical Group, Shiga, Japan), and 5 μl DNA extract. Negative (sterile water) and positive controls (DNA extract from the corresponding microorganism) were included in each run. The resulting amplicons were analysed on 2 % agarose gels containing 0.1 mg/ml ethidium bromide and visualised under UV light. Direct sequencing was performed on all positive PCR products by GATC Biotech (Cologne, Germany). Contiguous sequences were compared with known sequences listed in the GenBank nucleotide sequence database using the Blast search option of the National Center for Biotechnology Information (www.ncbi.nlm.nih.gov/BLAST).

### Statistical analysis

Initially we assessed the association of meteorological variables with the observed intra-annual variation in nymph density, by fitting each variable into a univariate log-linear regression analysis using Generalized Linear Mixed Models (GLMM) in a simplified approach to that we have previously used for forecasting dengue in Thailand [26]. Preliminary analyses showed that minimum temperature was always more significantly associated with nymph density and was thus used subsequently. The effects of average minimum monthly temperature (°C), numbers of current month frost or ice days, and precipitation (mm), on nymph density were analysed by log-linear regression using the Generalized Linear Mixed Model of GenStat version 15, with Month nested within Year fitted as a random factor. Wald statistics, which have a *χ*^2^ distribution, were established. Days were considered to be frost days when the minimum daily temperature fell below 0 °C. An ice day or permanent frosts were designated when even the highest daily temperature remained below 0 °C. Lagged monthly temperature and precipitation data were also examined for any lag effects on monthly nymph density. Lag effects were considered to be important because of the potential gradual depletion of the existing nymph stock [10]. All univariate analysis variables with a *P*-value ≤ 0.25 associated with nymph density were included in the multivariate analysis. Model simplification following fitting of the full model of the multivariate analysis was based on the change in the Akaike Information Criterion (AIC). Individual parameters were sequentially removed and then replaced and the new model that generated the largest drop in AIC, following removal of a variable, was retained. This was repeated until there was no further decrease in AIC. The standard rule of thumb for model comparisons suggests that a change in AIC of 2 or less from one model to the next means that the models are indistinguishable with regards to best fit. Here we additionally adopted a parsimonious approach such that, as can be seen in the Additional file 1, even if model simplification did not alter AIC greater than 2, we chose the model with the least number of parameters when accompanied by a decrease in AIC.

Secondly, in order to assess the relative contributions (to variation in nymph densities) of inter-annual variation in meteorological parameters and year per se, the percentage of variation in annual nymph densities explained by identified significant meteorological variables from the previous analysis and year, was assessed by log-linear regression in a GLMM. Year was thus designated as an explanatory factor, unlike the random factors above. Month was fitted as a random factor.

## Results

### Tick collection

From 2008 to 2014, a total of 8747 ticks were collected from the vegetation; of these 8594 were identified as *I. ricinus*. The remaining ticks were adult *Dermacentor reticulatus*, and were excluded from the study. The *I. ricinus* sample comprised 6294 nymphs and 2300 adults (1133 females and 1167 males) (Table 1).

DNA was extracted from 345 representative female ticks and 69 questing females in 2008 and 276 female carcasses after egg-laying that came from all months sampled within each year from 2009 to 2014. The *I. ricinus 16S rRNA* gene was amplified in 259/345 tick samples (75 %), which varied according to the year of collection (Table 2). Positive tick samples were then utilised to estimate pathogen prevalence.

**Table 2:** Prevalence of *Ixodes ricinus* female samples from Sénart Forest (France) that harbour DNA of selected micro-organisms from spring 2008 to spring 2014

Year	2008	2009	2010	2011	2012	2013	20
Number of ticks tested	69^a^	73	28	4	50	14	21
*I. ricinus* *16S* *rRNA* detection rate (%)	100	87	37	36	70	100	100
*A. phagocytophilum*	1 (1.5 %)	6 (8.2 %)	1 (3.6 %)	1 (25 %)	8 (16 %)	0	0
*Anaplasma* spp*.* ^b^	11 (16 %)	0	0	0	0	0	0
*M. mitochondrii*	51 (74 %)	1 (1.3 %)	0	0	0	0	0
*Rickettsia* spp*.*	5 (7.3 %)	1 (1.4 %)	0	0	0	0	0
*Babesia-Theileria* spp*.*	1 (1.5 %)	2 (2.7 %)	1 (3.6 %)	0	1 (2 %)	0	0
*Francisella tularensis*	1 (1.5 %)	0	0	0	0	0	0
*Borrelia burgdorferi* (*s.l*.)	20 (29 %)	0	0	0	0	0	0
*Borrelia miyamotoi*	2 (3 %)	0	0	0	0	0	0
*Bartonella* spp*.*	0	0	0	0	4 (8 %)	0	0

Detection was performed on questing females in 2008 and on post-laying female carcasses from 2009 to 2014

^a^Results from Reis et al. [22]

^b^Sequences obtained were all relative to uncultured *Anaplasma* spp. identified in ticks

### Tick density change over time

**Fig. 2:**
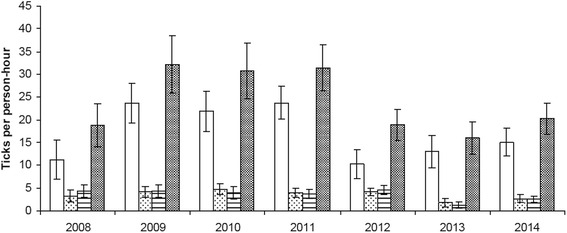
Annual variation in tick densities. Shown are means and standard errors of the number of ticks collected per person-hour. Histogram patterns: open rectangles, nymphs; sparse dots, adult females; horizontal lines, adult males; speckled, total number of ticks

**Fig. 3:**
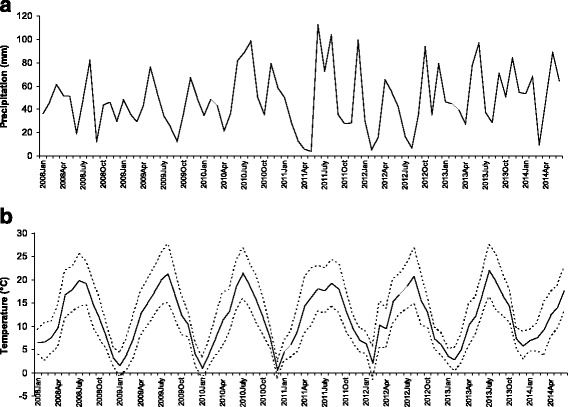
**a** Mean monthly precipitation. **b** Mean, minimum, and maximum monthly temperatures

Figure 2 shows the annual variation in tick densities (nymphs, adult females and adult males), with considerable inter-annual variation in observed nymph densities and no changes in adult tick populations. Figure 3a and b shows the annual variation in precipitation and ambient temperature per month. We first analysed the effect of meteorological variables on the observed intra-annual nymph variation, prior to addressing their potential contribution towards the inter-annual variation.

### Effect of meteorological variables on intra-annual nymph densities

**Fig. 4:**
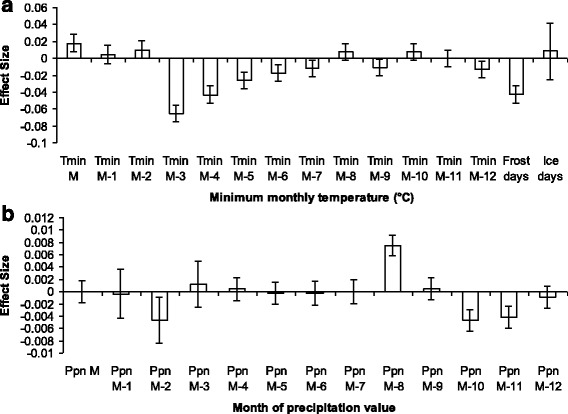
**a** Association of minimum monthly temperatures over the past year with nymph density at month M. *T*_min M_ is the minimum monthly temperature during the same month as the nymph density estimate; *T*_min M-1_ is the minimum monthly temperature 1 month before the nymph density estimate; *T*_min M-2_ is the minimum monthly temperature 2 months before, and so on. **b** Association of monthly precipitation values over the past year with nymph density at month M. M is the monthly precipitation during the same month as the nymph density estimate; M-1 is the monthly precipitation 1 month before the nymph density estimate; M-2 is the monthly precipitation 2 months before, and so on

Minimum monthly temperatures yielded better overall association with nymph density than either mean or maximum monthly temperatures (data not shown). Figure 4a, b demonstrates the effect sizes (which correspond to the beta coefficients of the parameters in the linear statistical model) of current and previous minimal monthly temperatures and precipitation in the univariate analyses. The current month’s minimum temperatures at the time of tick collection were positively associated with nymph density, whereas there was notably a lagged negative association with minimum temperatures in months 3 to 7 months previously. The number of current monthly frost days was negatively associated with current nymph density. There was a considerably weaker association of nymph densities with precipitation as compared to that observed with minimum temperature; effect sizes were all less than 0.01 and thus explained very little of the observed variation in nymph density.

In the final multivariate model, only monthly minimum temperature readings (current and lagged) and frost days were significantly associated with variation in nymph density. All effect sizes were small; only minimum temperature 10 months lagged had an effect size greater than 0.3 (Table 3). There was, therefore, no evidence for a strong effect of temperature, current or lagged, on monthly nymph densities.

**Table 3:** Meteorological variables associated with monthly nymph density in the minimal adequate model of the multivariate analysis

Variable	Effect size	Wald statistic	*P*-value
Tmin M	0.098	74.32	<0.001
Tmin M-2	-0.066	35.66	< 0.001
Tmin M-4	-0.127	116.81	< 0.001
Tmin M-5	-0.255	577.33	< 0.001
Tmin M-7	0.135	154.9	< 0.001
Tmin M-8	0.263	414.25	< 0.001
Tmin M-9	-0.251	536.37	< 0.001
Tmin M-10	-0.306	757.73	< 0.001
Frost days	-0.287	640.61	< 0.001

*Abbreviations*: *Tmin* minimum monthly temperature; *Tmin M-2* minimum monthly temperature 2 months previously; *Tmin M-4* minimum monthly temperature 4 months previously; *Tmin M-5* minimum monthly temperature 5 months previously; *Tmin M-7* minimum monthly temperature 7 months previously; *Tmin M-8* minimum monthly temperature 8 months previously; *Tmin M-9* minimum monthly temperature 9 months previously; *Tmin M-10* minimum monthly temperature 10 months previously; *Frost days* current month’s number of frost days

### Effect of meteorological variables on inter-annual densities

Only the meteorological variables *T*_min M-8_, *T*_min M-9_, and current month’s frost days explained any significant annual variation in nymph densities (0.1, 7 and 27 % respectively). Year explained 59 % of the variation (Table 4).

**Table 4:** Association of year and meteorological variables with monthly nymph density in the minimal adequate model of the multivariate analysis

Variable	Wald statistic	Degrees of freedom	*P*-value	Percent of variation explained
Year	53.41	6	0.004	58.7
Tmin M-8	24.53	1	0.002	0.1
Tmin M-9	23.72	1	0.002	6.8
Frost days	21.92	1	0.002	26.7

*Abbreviations*: *Tmin M-8* minimum monthly temperature 8 months previously; *Tmin M-9* minimum monthly temperature 9 months previously

### Pathogenic DNA in ticks

PCR results from the 259 tested females showed variable yearly prevalence rates (Table 2) and an overall 7-year prevalence of 10.8 % for *Anaplasma* spp.; 2.3 % for SFG *Rickettsia* spp.; 2 % for *Babesia/Theileria* spp.; 0.4 % for *F. tularensis*; 1.4 % for *Bartonella* spp.; 7.7 % for *B. burgdorferi* (*s.l*.); 0.7 % for *B. miyamotoi*; 1.5 % for *Bartonella* spp.; and 20 % *Ca.* M. mitochondrii (Table 2). The following pathogen species were then identified: *Anaplasma phagocytophilum*, *Rickettsia helvetica*, *Babesia venatorum* and *B. divergens*, *F. tularensis*, *B. miyamotoi*, *Borrelia afzelii/valaisiana*, and *Borrelia garinii/lusitaniae* (amplified sequences were not sufficiently discriminatory to permit distinction between *B. garinii* and *B. lusitaniae*, or between *B. afzelii* and *B. valaisiana*), and the tick symbiont *Ca.* M. mitochondrii. Only one tick collected in 2010 was positive for two pathogens, *A. phagocytophilum* and *B. divergens.* However, as we were not able to differentiate *B. afzelii* and *B. valaisiana*, as well as *B. garinii* and *B. lusitaniae*, a co-infection by various *Borrelia* spp. cannot be excluded.

## Discussion

Our 7-year longitudinal survey revealed fluctuating densities in populations of nymphs, but no changes in adult tick populations. It is well known that abiotic factors, such as temperature and humidity, are key determinants in tick development, survival, and activity [27–31]. There were a number of associations between meteorological factors and variation in annual/monthly nymph density. First, minimum and not mean or maximum monthly temperatures were more strongly associated with nymph density. These results are consistent with the recognised temperature-dependent activity of *I. ricinus* in France, where activity occurs from ~7 °C, with an upper limit, which is influenced by epicuticle degradation that occurs at 32 °C and mortality at 40 °C, beyond that observed in our study (27.7 °C) [32]. This is again reflected in the positive association with current monthly temperatures (and negative association with the number of frost days that month); current temperatures above 7 °C increasingly induce questing behavior by the nymph population up to a limit (~20 °C) at which point there is no further incremental increase in activity [32].

The weak and opposing associations of *I. ricinus* densities with temperatures recorded 8 to 10 months earlier is intriguing, but must be treated with caution. The nymph densities in our study were measured from March to June; 8 to 10 month temperature lags would correspond to the months of May to November the previous year. The positive (8 months lag) and negative (9 and 10 months lag) associations of minimum temperature with nymph density may be an artifact arising from the seasonal dynamics of nymph densities. However, it is notable, irrespective of the direction of the effect, that these lagged months have a stronger association with nymph density than does the current month’s temperature. Complex, longer term effects of temperature on tick population dynamics have been noted before. Warmer autumnal temperatures may increase tick survival/developmental rates, leading to increased nymphal densities the following summer. Indeed, Lauterbach et al. [13] have demonstrated that nymph density was strongly linked to the preceding winter temperatures, with higher tick densities associated with milder weather conditions. Alternatively, milder autumnal temperatures and lower nymph abundance the following spring-summer could be due to more newly-molted nymphs successfully feeding in autumn, thereby depleting the active nymph stock prior to the following spring. Monthly collections occurring throughout the year are the only effective way to measure this effect. Despite some indication that minimum temperature several months ago is associated with monthly nymph density, effect sizes are generally small and there is no single lagged month Tmin that enables us to pinpoint a strong association and hence generate a forecast of future nymph densities.

In contrast to the intra-annual variation, only the number of current frost days had any notable impact on inter-annual densities, clearly reflecting the lower temperature limit of nymph activity. Low inter-annual variation in temperature and limited tick sampling may have yielded insufficient resolution for detecting any such influences of temperature on nymph density beyond the “extreme” temperature variable of “frost days”. Significant impact of extreme weather variables has been noted before as being determinant in influencing inter-annual densities [15]. Nevertheless, the majority of the inter-annual variation was explained by the year per se. One cause behind such yearly variation may concern variation in vertebrate host species abundance; the ease with which ticks have access to vertebrate host species is a major recognised factor affecting tick densities [33, 34]. From 2005 to 2010, the deer population in the Domaine de la Faisanderie, a specific closed region of the forest, increased four-fold from 0.2 to 1 deer per hectare, and subsequently suffered a population crash during the winter of 2010–2011, dropping back to half their numbers (J-L Chapuis, personal communication). In addition, a sharp decline in the rodent population was observed in 2009 in this forest and which only started to recover in 2014 (J-L Chapuis, personal communication). Tick larvae are known to feed on rodents and nymphs and adults on larger mammals and thus the reduced host population likely contributed to the observed variation in nymph densities, as previously described [5].

Pathogen prevalence rates varied independently of nymph or total tick densities and different pathogens showed very different patterns over time. *Borrelia* spp. was the most prevalent identified pathogen in 2008 (32 %), but surprisingly, was not detected at all over the following years. In a previous 2008 study of the same area, the prevalence of this pathogen was determined to be 20 % for nymphal and adult ticks [22], and other studies have published 0 to 39 % *Borrelia* prevalence in *I. ricinus* females from France [35–41]. The observed disappearance of *Borrelia* spp. may be due to the decrease in rodent and chipmunk reservoir populations mentioned above [42, 43]. Similarly, *Rickettsia* spp. were only detected in 2008 and 2009. All derived *Rickettsia* sequences were related to *R. helvetica*, which is strongly suspected to be transmitted by *I. ricinus.* In 2008, the prevalence of *R. helvetica* (7.3 %) was similar to that reported from studies in central France (8.7 %) [38], whereas the prevalence in 2009 (1.4 %) was similar to data from western France (1.4 %) [40]. However, the apparent disappearance of both *Borrelia* spp. and *R. helvetica* should be interpreted with caution for two reasons. First, because of some unsuccessful tick engorgements some years, the number of ticks PCR-screened for pathogens each year fluctuated widely (from four in 2011, to 73 in 2009), and thus was occasionally too low to enable informative comparisons between years. The relatively small number of samples analysed in 2013 and 2014 could be an explanation for the apparent absence of pathogens in the tick population. Secondly, DNA extracted from post-lay adult female carcasses from 2009 to 2014 seemed to be more unpredictable than that extracted from questing females, as the *I. ricinus 16SrRNA* detection rate varied from 36 to 100 % between the years of 2009 to 2014, compared to 100 % obtained from questing ticks in 2008.

Unlike *Borrelia* spp., *A. phagocytophilum* was detected from 2008 to 2012, with prevalence rates that seemed to increase from 1.5 to 16 %, taking into account the potential caveat mentioned above. These rates are similar to those reported from other areas in France, which range from 0.3 to 23.7 % [38, 40, 44–47]. *Babesia* spp. were also detected in 2008, 2009, 2010 and 2013, with prevalence rates varying from 1.5 to 3.6 %, again reflecting other prevalence rates reported in Europe for *I. ricinus* [48–51]. All sequences were related to *B. venatorum* except one that corresponded to *B. divergens.* Both in vivo and in vitro studies have identified roe deer as a potential wild reservoir for *B. venatorum* and *I. ricinus* as the vector [52–54]. Detecting *B. divergens* in an area without cattle reinforces the hypothesis that wild reservoirs for this parasite do exist [4]. In addition to *Babesia microti*, these two zoonotic parasites are responsible for human cases of babesiosis (see review by [55]).

Both *F. tularensis* and *Bartonella* spp. were only detected at one time point during the study, in 2008 and 2012, respectively. Small animals such as rabbits, hares, voles, and muskrats serve as reservoir hosts for *F. tularensis*, and cases of tularemia can occur throughout the entire northern hemisphere. Currently, the disease is thought to be rare and limited to hares and hunters, with tick transmission considered trivial in the natural cycle of transmission [56]. These circumstances are likely identical for *Bartonella* spp., for which transmission by ticks was strongly debated for several years, even though *I. ricinus* competency has now been demonstrated both in vitro [57] and in vivo [58]. A previous study performed in the same area identified *Bartonella birtlesii* in nymph pools [22], but we failed to sequence the detected species in the present study.

## Conclusions

We reconfirm that *I. ricinus* is an important vector of pathogenic microorganisms, not only in woodland areas, but also in suburban forests, which is of public health relevance due to the potentially frequent exposure of humans and domestic animals to possibly infected ticks. Our results also suggest that despite complex intra-annual relationships between tick densities and abiotic factors, there is no evidence for a climate-associated increase in infection risk over the 7-year period. Although both tick and vertebrate host populations can be influenced by variation in climate factors, the lack of variation in abiotic conditions between years in our study may have precluded detection of any impact on tick densities. The inter-annual fluctuations in tick densities are likely to be strongly influenced by population density fluctuations in vertebrate host species and then wildlife management. Whilst climate change is predicted to affect the distribution of tick species [59, 60], studies on the relative influence of meteorological factors on tick-borne diseases in currently endemic areas needs to be addressed further using larger studies with finer scale measurements of abiotic variables.

## Additional file

Additional file 1: Model reduction procedure using AIC. (DOC 38 kb)

## References

[1] Dantas-Torres F, Chomel BB, Otranto D (2012). Ticks and tick-borne diseases: a one health perspective. Trends Parasitol.

[2] Madder M, Thys E, Achi L, Toure A, De Deken R (2011). *Rhipicephalus* (*Boophilus*) *microplus*: a most successful invasive tick species in West-Africa. Exp Appl Acarol.

[3] Vayssier-Taussat M, Moutailler S, Michelet L, Devillers E, Bonnet S, Cheval J, Hebert C, Eloit M (2013). Next generation sequencing uncovers unexpected bacterial pathogens in ticks in western Europe. PLoS One.

[4] Bonnet S, Michelet L, Moutailler S, Cheval J, Hebert C, Vayssier-Taussat M, Eloit M (2014). Identification of parasitic communities within European ticks using next-generation sequencing. PLoS Negl Trop Dis.

[5] Leger E, Vourc’h G, Vial L, Chevillon C, McCoy KD (2013). Changing distributions of ticks: causes and consequences. Exp Appl Acarol.

[6] Lindgren E, Andersson Y, Suk JE, Sudre B, Semenza JC (2012). Public health. Monitoring EU emerging infectious disease risk due to climate change. Science.

[7] Heyman P, Cochez C, Hofhuis A, van der Giessen J, Sprong H, Porter SR, Losson B, Saegerman C, Donoso-Mantke O, Niedrig M (2010). A clear and present danger: tick-borne diseases in Europe. Expert Rev Anti Infect Ther.

[8] Rizzoli A, Silaghi C, Obiegala A, Rudolf I, Hubálek Z, Földvári G, Plantard O, Vayssier-Taussat M, Bonnet S, Špitalská E, et al. *Ixodes ricinus* and its transmitted pathogens in urban and peri-urban areas in Europe: new hazards and relevance for public health. Front Public Health. 2014;2:251. 10.3389/fpubh.2014.00251 PMC4248671 25520947

[9] Medlock JM, Hansford KM, Bormane A, Derdakova M, Estrada-Pena A, George JC, Golovljova I, Jaenson TG, Jensen JK, Jensen PM (2013). Driving forces for changes in geographical distribution of *Ixodes ricinus* ticks in Europe. Parasit Vectors.

[10] Vassallo M, Paul RE, Perez-Eid C (2000). Temporal distribution of the annual nymphal stock of *Ixodes ricinus* ticks. Exp Appl Acarol.

[11] Jouda F, Perret JL, Gern L (2004). Density of questing *Ixodes ricinus* nymphs and adults infected by *Borrelia burgdorferi* sensu lato in Switzerland: spatio-temporal pattern at a regional scale. Vector Borne Zoonotic Dis.

[12] Schwarz A, Maier WA, Kistemann T, Kampen H (2009). Analysis of the distribution of the tick *Ixodes ricinus* L. (Acari: Ixodidae) in a nature reserve of western Germany using Geographic Information Systems. Int J Hyg Environ Health.

[13] Lauterbach R, Wells K, O’Hara RB, Kalko EK, Renner SC (2013). Variable strength of forest stand attributes and weather conditions on the questing activity of *Ixodes ricinus* ticks over years in managed forests. PLoS One.

[14] Burri C, Moran Cadenas F, Douet V, Moret J, Gern L (2007). *Ixodes ricinus* density and infection prevalence of *Borrelia burgdorferi* sensu lato along a North-facing altitudinal gradient in the Rhone Valley (Switzerland). Vector Borne Zoonotic Dis.

[15] Daniel M, Malý M, Danielová V, Kříž B, Nuttall P (2015). Abiotic predictors and annual seasonal dynamics of *Ixodes ricinus*, the major disease vector of Central Europe. Parasit Vectors.

[16] Eisen L, Eisen RJ, Lane RS (2002). Seasonal activity patterns of *Ixodes pacificus* nymphs in relation to climatic conditions. Med Vet Entomol.

[17] Eisen RJ, Eisen L, Ogden NH, Beard CB (2016). Linkages of weather and climate with *Ixodes scapularis* and *Ixodes pacificus* (Acari: Ixodidae), enzootic transmission of *Borrelia burgdorferi*, and Lyme Disease in North America. J Med Entomol.

[18] Eisen RJ, Eisen L, Lane RS (2006). Predicting density of *Ixodes pacificus* nymphs in dense woodlands in Mendocino County, California, based on geographic information systems and remote sensing versus field-derived data. Am J Trop Med Hyg.

[19] Maresca B, Poquet G, Martin O. Enquete de frequentation dans la forêt de senart et dans les forêts domaniales des hauts-de-seine. Crédoc. 2005;vol. 3.

[20] Vassallo M, Pichon B, Cabaret J, Figureau C, Perez-Eid C (2000). Methodology for sampling questing nymphs of *Ixodes ricinus* (Acari: Ixodidae), the principal vector of Lyme disease in Europe. J Med Entomol.

[21] Pérez-Eid C (2007). Les Tiques : Identification, biologie, importance médicale et vétérinaire, Tec & Doc Lavoisier.

[22] Reis C, Cote M, Paul RE, Bonnet S (2011). Questing ticks in suburban forest are infected by at least six tick-borne pathogens. Vector Borne Zoonotic Dis.

[23] Bonnet SI, Liu XY (2012). Laboratory artificial infection of hard ticks: a tool for the analysis of tick-borne pathogen transmission. Acariologia.

[24] Halos L, Jamal T, Vial L, Maillard R, Suau A, Le Menach A, Boulouis HJ, Vayssier-Taussat M (2004). Determination of an efficient and reliable method for DNA extraction from ticks. Vet Res.

[25] Black WC, Piesman J (1994). Phylogeny of hard- and soft-tick taxa (Acari: Ixodida) based on mitochondrial 16S rDNA sequences. Proc Natl Acad Sci U S A.

[26] Lowe R, Cazelles, B, Paul R, Rodo X. Quantifying the added value of interannual climate variability in a spatio-temporal dengue model. Stoch Environ Res Risk Assess*.* 2015;1–12.

[27] Perret JL, Guigoz E, Rais O, Gern L (2000). Influence of saturation deficit and temperature on *Ixodes ricinus* tick questing activity in a Lyme borreliosis-endemic area (Switzerland). Parasitol Res.

[28] Hubalek Z, Halouzka J, Juricova Z (2003). Host-seeking activity of ixodid ticks in relation to weather variables. J Vector Ecol.

[29] Ogden NH, Maarouf A, Barker IK, Bigras-Poulin M, Lindsay LR, Morshed MG, O’Callaghan CJ, Ramay F, Waltner-Toews D, Charron DF (2006). Climate change and the potential for range expansion of the Lyme disease vector *Ixodes scapularis* in Canada. Int J Parasitol.

[30] Salkeld DJ, Castro MB, Bonilla D, Kjemtrup A, Kramer VL, Lane RS, Padgett KA (2014). Seasonal activity patterns of the western black-legged tick, *Ixodes pacificus*, in relation to onset of human Lyme disease in northwestern California. Ticks Tick Borne Dis.

[31] Padgett KA, Lane RS (2001). Life cycle of *Ixodes pacificus* (Acari: Ixodidae): timing of developmental processes under field and laboratory conditions. J Med Entomol.

[32] Vassallo-Paul M. Borréliose de Lyme : étude des densités du vecteur principal, le groupe d*’*espèces *Ixodes ricinus*, et de ses relations à l*’*environnement. Evaluation des zones et des périodes à risques pour l*’*homme de contracter la maladie, Thèse de doctorat de biologie, Université Pierre et Marie Curie. Paris, France. 2000;125 p.

[33] LoGiudice K, Duerr ST, Newhouse MJ, Schmidt KA, Killilea ME, Ostfeld RS (2008). Impact of host community composition on Lyme disease risk. Ecology.

[34] Dobson ADM, Randolph SE (2011). Modelling the effects of recent changes in climate, host density and acaricide treatments on population dynamics of *Ixodes ricinus* in the UK. J Appl Ecol.

[35] Randolph SE (2001). The shifting landscape of tick-borne zoonoses: tick-borne encephalitis and Lyme borreliosis in Europe. Philos Trans R Soc Lond B Biol Sci.

[36] Beytout J, George JC, Malaval J, Garnier M, Beytout M, Baranton G, Ferquel E, Postic D (2007). Lyme borreliosis incidence in two French departments: correlation with infection of *Ixodes ricinus* ticks by *Borrelia burgdorferi* sensu lato. Vector Borne Zoonotic Dis.

[37] Halos L, Jamal T, Maillard R, Beugnet F, Le Menach A, Boulouis HJ, Vayssier-Taussat M (2005). Evidence of *Bartonella* sp. in questing adult and nymphal *Ixodes ricinus* ticks from France and co-infection with *Borrelia burgdorferi* sensu lato and *Babesia* sp. Vet Res.

[38] Halos L, Bord S, Cotte V, Gasqui P, Abrial D, Barnouin J, Boulouis HJ, Vayssier-Taussat M, Vourc’h G (2010). Ecological factors characterizing the prevalence of bacterial tick-borne pathogens in *Ixodes ricinus* ticks in pastures and woodlands. Appl Environ Microbiol.

[39] Zhioua E, Postic D, Rodhain F, Perez-Eid C (1996). Infection of *Ixodes ricinus* (Acari:Ixodidae) by *Borrelia burgdorferi* in Ile de France. J Med Entomol.

[40] Cotté V, Bonnet S, Cote M, Vayssier-Taussat M (2010). Prevalence of five pathogenic agents in questing *Ixodes ricinus* ticks from western France. Vector Borne Zoonotic Dis.

[41] Bonnet S, de la Fuente J, Nicollet P, Liu X, Madani N, Blanchard B, Maingourd C, Alongi A, Torina A, Fernandez de Mera IG (2013). Prevalence of tick-borne pathogens in adult *Dermacentor* spp. ticks from nine collection sites in France. Vector Borne Zoonotic Dis.

[42] Marsot M, Chapuis JL, Gasqui P, Dozieres A, Masseglia S, Pisanu B, Ferquel E, Vourc’h G (2013). Introduced Siberian chipmunks (*Tamias sibiricus barberi)* contribute more to lyme borreliosis risk than native reservoir rodents. PLoS One.

[43] Marsot M, Sigaud M, Chapuis JL, Ferquel E, Cornet M, Vourc’h G (2011). Introduced Siberian chipmunks (*Tamias sibiricus barberi*) harbor more-diverse *Borrelia burgdorferi* sensu lato genospecies than native bank voles (*Myodes glareolus*). Appl Environ Microbiol.

[44] Grzeszczuk A, Stanczak J (2006). High prevalence of *Anaplasma phagocytophilum* infection in ticks removed from human skin in north-eastern Poland. Ann Agric Environ Med.

[45] Sanogo YO, Parola P, Shpynov S, Camicas JL, Brouqui P, Caruso G, Raoult D (2003). Genetic diversity of bacterial agents detected in ticks removed from asymptomatic patients in northeastern Italy. Ann N Y Acad Sci.

[46] Halos L, Vourc’h G, Cotte V, Gasqui P, Barnouin J, Boulous HJ, Vayssier-Taussat M (2006). Prevalence of *Anaplasma phagocytophilum, Rickettsia* sp. and *Borrelia burgdorferi* sensu lato DNA in questing *Ixodes ricinus* ticks from France. Ann N Y Acad Sci.

[47] Parola P, Beati L, Cambon M, Brouqui P, Raoult D (1998). Ehrlichial DNA amplified from *Ixodes ricinus* (Acari: Ixodidae) in France. J Med Entomol.

[48] Duh D, Petrovec M, Avsic-Zupanc T (2005). Molecular characterization of human pathogen *Babesia* EU1 in *Ixodes ricinus* ticks from Slovenia. J Parasitol.

[49] Casati S, Sager H, Gern L, Piffaretti JC (2006). Presence of potentially pathogenic *Babesia* sp. for human in *Ixodes ricinus* in Switzerland. Ann Agric Environ Med.

[50] Cieniuch S, Stanczak J, Ruczaj A (2009). The first detection of *Babesia* EU1 and *Babesia canis* canis in *Ixodes ricinus* ticks (Acari, Ixodidae) collected in urban and rural areas in northern Poland. Pol J Microbiol.

[51] Wielinga PR, Fonville M, Sprong H, Gaasenbeek C, Borgsteede F, Giessen JW (2008). Persistent detection of *Babesia* EU1 and *Babesia microti* in *Ixodes ricinus* in the Netherlands during a 5-year surveillance: 2003-2007. Vector Borne Zoonotic Dis.

[52] Bonnet S, Jouglin M, Malandrin L, Becker C, Agoulon A, L’Hostis M, Chauvin A (2007). Transstadial and transovarial persistence of *Babesia divergens* DNA in *Ixodes ricinus* ticks fed on infected blood in a new skin-feeding technique. Parasitology.

[53] Becker CA, Bouju-Albert A, Jouglin M, Chauvin A, Malandrin L (2009). Natural transmission of Zoonotic *Babesia* spp. by *Ixodes ricinus* ticks. Emerg Infect Dis.

[54] Bonnet S, Brisseau N, Hermouet A, Jouglin M, Chauvin A (2009). Experimental *in vitro* transmission of *Babesia* sp. (EU1) by *Ixodes ricinus*. Vet Res.

[55] Gray J, Zintl A, Hildebrandt A, Hunfeld KP, Weiss L (2010). Zoonotic babesiosis: overview of the disease and novel aspects of pathogen identity. Ticks Tick Borne Dis.

[56] Foley JE, Nieto NC (2009). Tularemia. Vet Microbiol.

[57] Cotte V, Bonnet S, Le Rhun D, Le Naour E, Chauvin A, Boulouis HJ, Lecuelle B, Lilin T, Vayssier-Taussat M (2008). Transmission of *Bartonella henselae* by *Ixodes ricinus*. Emerg Infect Dis.

[58] Reis C, Cote M, Le Rhun D, Lecuelle B, Levin ML, Vayssier-Taussat M, Bonnet SI (2011). Vector competence of the tick *Ixodes ricinus* for transmission of *Bartonella birtlesii*. PLoS Negl Trop Dis.

[59] Qviller L, Grova L, Viljugrein H, Klingen I, Mysterud A (2014). Temporal pattern of questing tick *Ixodes ricinus* density at differing elevations in the coastal region of western Norway. Parasit Vectors.

[60] Lindgren E, Talleklint L, Polfeldt T (2000). Impact of climatic change on the northern latitude limit and population density of the disease-transmitting European tick *Ixodes ricinus*. Environ Health Perspect.

