# Supplementary material for: Environmental factors influencing tick densities over seven years in a French suburban forest
Source: Parasit Vectors. 2016 May 27;9:309. doi: 10.1186/s13071-016-1591-5 (PMC4884405; doi:10.1186/s13071-016-1591-5)
Supplement: Additional file 1: — Model reduction procedure using AIC. (DOC 38 kb) [file 13071_2016_1591_MOESM1_ESM.doc]

**Model reduction procedure using AIC**

All Monthly minimum Temperatures (current month or lagged) that were significant at P≤0.25 in the univariate anlaysis on their effect on monthly nymph densities were combined in a multivariate analysis. Model simplification was then performed by repeated removal and replacement of variables one by one and the variable whose removal led to the largest decrease in AIC was then removed. This was repeated until removal of any of the remaining variables led to no further decrease in AIC. The standard rule of thumb for model comparisons suggests that a change in AIC of 2 or less from one model to the next means that the models are indistinguishable with regards to best fit. Here we additionally adopted a parsimonious approach such that, as can be seen in the table below, even if model simplification did not alter AIC greater than 2, we chose the model with the least number of parameters when accompanied by a decrease in AIC.

Then monthly precipitation variables (current and lagged) found to be significant at P≤0.25 in the univariate anlaysis were added and the process repeated.

| Temperature univariate P≤0.25 months | | | | | | |
| --- | --- | --- | --- | --- | --- | --- |
| Model reduction based on AIC | | | |  |  |  |
|  |  | | AIC value |  |  |  |
| Full T min model | | -24.64 | |  |  |  |
| Remove T Min-6 | | -32.66 | |  |  |  |
| Remove T Min-3 | | -38.67 | |  |  |  |
| Remove Tmin-12 | | -38.73 | |  |  |  |
|  |  | | This is lowest AIC | |  |  |
| Addition of univariate P≤0.25 ppn parameters | | | | | | |
|  |  | |  |  |  |  |
| Full model |  | | 62.12 |  |  |  |
| Remove ppn-10 | | 49.03 | |  |  |  |
| Remove ppn-11 | | 37.2 | |  |  |  |
| Remove ppn-8 | | 29.07 | |  |  |  |
| Remove ppn-2 | | -38.73 | |  |  |  |
|  |  | |  |  |  |  |
|  | Therefore ppn parameters do not provdie additional explanatory power | | | | | |
|  | Their effect sizes are all less than 0.01 | | | | | |
